# Accreditation council for graduate medical education (ACGME) annual anesthesiology residency and fellowship program review: a "report card" model for continuous improvement

**DOI:** 10.1186/1472-6920-10-13

**Published:** 2010-02-08

**Authors:** Steven H Rose, Timothy R Long

**Affiliations:** 1 Department of Anesthesiology, College of Medicine, Mayo Clinic, Rochester, MN 55905, USA

## Abstract

**Background:**

The Accreditation Council for Graduate Medical Education (ACGME) requires an annual evaluation of all ACGME-accredited residency and fellowship programs to assess program quality. The results of this evaluation must be used to improve the program. This manuscript describes a metric to be used in conducting ACGME-mandated annual program review of ACGME-accredited anesthesiology residencies and fellowships.

**Methods:**

A variety of metrics to assess anesthesiology residency and fellowship programs are identified by the authors through literature review and considered for use in constructing a program "report card."

**Results:**

Metrics used to assess program quality include success in achieving American Board of Anesthesiology (ABA) certification, performance on the annual ABA/American Society of Anesthesiology In-Training Examination, performance on mock oral ABA certification examinations, trainee scholarly activities (publications and presentations), accreditation site visit and internal review results, ACGME and alumni survey results, National Resident Matching Program (NRMP) results, exit interview feedback, diversity data and extensive program/rotation/faculty/curriculum evaluations by trainees and faculty. The results are used to construct a "report card" that provides a high-level review of program performance and can be used in a continuous quality improvement process.

**Conclusions:**

An annual program review is required to assess all ACGME-accredited residency and fellowship programs to monitor and improve program quality. We describe an annual review process based on metrics that can be used to focus attention on areas for improvement and track program performance year-to-year. A "report card" format is described as a high-level tool to track educational outcomes.

## Background

The Accreditation Council for Graduate Medical Education (ACGME) requires an annual evaluation of all ACGME-accredited residency and fellowship programs[1]. This annual program evaluation must document formal, systematic evaluation of the curriculum and must monitor and track resident performance, faculty development, graduate performance (including performance of program graduates on their respective certification examinations) and program quality. Assessment of program quality must provide residents and faculty the opportunity to evaluate the program confidentially and in writing at least annually. These assessments of the program, together with other program evaluation results, must be used to improve the program. If deficiencies are identified, a written plan of action must be generated to document initiatives to improve program performance. The action plan must be reviewed and approved by the teaching faculty and documented in meeting minutes. Although there have been several publications that address a graduate medical education (GME) "score card" to compare residencies in various specialties within a sponsoring institution, we were unable to identify publications that specifically addressed the ACGME-required annual program review in anesthesiology [2-6].

We describe a systematic annual review process for assessing ACGME-accredited anesthesiology residency and fellowship programs that meets ACGME requirements, includes metrics that can be used to assess program quality and provides a model for continuous quality improvement.

## Methods

A literature search was initiated to identify metrics that might be used in this process and each metric identified in the previously published articles we reviewed was considered for inclusion in our annual program assessment. Criteria for inclusion included the ability to accurately access and collect the data with a high level of confidence and the authors' agreement the metric was important in the assessment of program quality. Metrics to be included were also reviewed by our department education committee and a consensus was generated regarding each item. Some previously described metrics, such as the ratio between the number of persons matched through the National Resident Matching Program (NRMP) and the last rank number at which the program filled (rank/match ratio), were eliminated due to concerns regarding the ability to manipulate these data.

## Results

Metrics selected for inclusion in the annual review of our ACGME-accredited anesthesiology residency and fellowship programs are listed in Table 1. Each metric included in this review is further described, including the source(s) of the data, below:

**Table 1:** Annual Program Review Metrics

A. Examination performance
1. Five-year ABA certification examination results
2. CA-3 resident performance on the ASA/ABA In-Training Examination
3. CA-3 resident performance on program-administered mock oral examination
B. Resident Scholarly Activities
1. Percent of residents with one or more manuscripts completed, submitted, accepted for publication or published based on work completed during the program
2. Percent of residents with one or more posters/abstracts completed, submitted or published based on work completed during the program
3. Percent of residents with one or more regional, national or international presentation while enrolled in the program

C. Accreditation and Internal Reviews
1. ACGME site visit interval
2. ACGME site visit citations
3. Number of Internal Review suggestions/citations not fully addressed within six months

D. ACGME Survey
1. Number of items with greater than 10% non-compliant response rate on ACGME resident survey

E. NRMP Results
1. Mean score on USMLE Step 1 for matched residents
2. Mean score on USMLE Step 2 for CA-2 residents
3. "Filling" through NRMP
4. Post NRMP match survey results

F. Exit interview assessments

G. Case/procedure numbers

H. Diversity
1. Data for women compared to national averages
2. Data for under-represented minorities compared to national averages

I. Alumni survey results

J. ISES evaluation results
1. Resident evaluations
a. program
b. rotations
c. curriculum
d. faculty
2. Faculty evaluations
a. program
b. curriculum
c. residents

### Examination Performance

Performance of graduates on the American Board of Anesthesiology (ABA) certification examinations. The ACGME explicitly uses this metric to assess program outcomes[1]. Arguably, it is the best single measure of program outcomes currently available. The ACGME and Anesthesiology Review Committee consider five-year board certification passage rates as an important indicator of program quality when the program is reviewed for accreditation. Anesthesiology residency programs and those anesthesiology fellowship programs that grant certificates of special qualifications can review historical results using this metric, establish goals for five-year board certification passage rates and track performance year-to-year. Performance below expected standards prompts a review and a written plan of action to improve outcomes.

Clinical Anesthesia-3 (CA-3) resident performance on the ABA/American Society of Anesthesiology (ASA) In-Training Examination (ITE) can provide a more contemporary assessment of preparedness for the ABA written certification examination. Our program established expected standards for individual and collective CA-3 performance on the ITE and compares these results to national standards. Performance below expectations on the ITE prompts a review and a written plan of action to improve outcomes.

CA-3 resident performance on regularly scheduled mock oral examinations provides an assessment of PGY-level specific preparedness for the ABA oral certification examination. Consistency regarding expected standards for CA-3 performance on program-administered mock ABA oral examinations was established through a faculty development process. To make this process better approximate the ABA oral certification examination, a senior or emeritus faculty member acts as a docent, retired ABA oral board examination questions are used for the examination and the examination is conducted in the exact format of the ABA oral board certification examination. The only major difference is that time is scheduled after the examination to debrief and provide resident feedback. Expectations for CA-3 resident performance are at a level consistent with passing the examination when taken for credit. Performance at a marginal or failing level prompts a review, requires a written plan of action and requires additional practice examinations to improve performance.

### Resident Scholarly Activities

Resident publications in peer-reviewed journals are included in our annual review as a metric assessing program quality. In order to be included in this metric, the resident must have completed the work resulting in publication during his or her residency training. To assess program performance in resident publication in greater detail, we record additional information including the order of authorship and the impact factor of the journal in which the manuscript is published. Resident publication in non-peer reviewed journals and publication of chapters are considered separately.

The number of posters and abstracts completed, accepted and published are similarly recorded.

Scholarly activity metrics also include an assessment of resident presentations at regional, national and international meetings.

### Accreditation and Internal Reviews

ACGME accreditation outcomes are a key metric in the annual review of our ACGME-accredited residency and fellowship programs. Our programs' goal and expectation for site visit interval is five years. Performance below this standard requires generation of an action plan.

Review Committee citations are also monitored as a metric of program quality. We have established two or fewer citations as our programs' goal. Regardless of the total number, an action plan and follow-up report are required to address each citation.

Internal reviews (IR). The ACGME requires the Sponsoring Institution to conduct an internal review of each program at the midpoint of the accreditation cycle. This is a separate review that contains distinct elements. If the program receives a five-year accreditation cycle, an internal review is only conducted once every five years. Internal reviews are handled in a manner similar to that used to assess accreditation results. The number of IR suggestions is tracked and all IR suggestions must be addressed in writing, including an action plan to address any concerns. A follow-up report is required to assure the action plan has been implemented and improvement has occurred. Our programs' goal is to have all IR suggestions addressed and corrected within six months. We do not consider the total number of recommendations in the internal review as a metric since the internal review process is a valued mechanism for feedback intended to improve the program and recommendations are encouraged.

### ACGME Resident Survey

The anonymous ACGME resident survey is a relatively new metric that can be used to assess program quality and duty hour compliance. We chose ACGME survey results as a key metric in our annual program review due to the confidence residents have in the anonymity of the process. However, the authors recognize survey data may be influenced by a variety of factors including concern about an adverse accreditation action and/or administrative pressure. Duty hours are also measured by resident attestation and duty hour violations can be reported on an anonymous MSGME school "hotline" and/or anonymously reported to our residents association. Non-compliance of greater than 10% on any item requires a written action plan to address the issue(s) and a follow-up report to assure the concern has been addressed and corrected.

### NRMP Results

The number of applications, invitations to interview and interviews conducted are tracked on an annual basis and the outcomes of the match are analyzed. Although the value of standardized examinations in assessing resident quality is limited, we believe most programs consider standardized examination performance as an important metric when selecting residents. As such, they are an indirect measure of the competitiveness of our program related to others. We have established a program goal of 220 for the mean score of matched applicants on Step 1 of the United States Medical Licensing Examination (USMLE) and average USMLE scores are compared year-to-year.

Goals can also be established for matched applicant scores on step 2 of the USMLE. Although the content of the questions on step 2 of the USMLE is more directly related to clinical medicine, the immediate value of this metric is lessened because not all applicants receive the results of this examination in time to influence their position on the NRMP rank order list, which probably results in self-selection bias. To address this issue, USMLE step 2 scores for PGY 2 residents (when all should be available) are reviewed and included in our annual program review.

"Filling" through the NRMP each year is a goal of our program. Although NRMP result analysis and outcomes can be used to establish a rank/match ratio as an index of performance in attracting highly-qualified applicants to the program, this metric is subject to manipulation (residents can be ranked on their likelihood to choose the program, rather than on their desirability). Consequently, this metric is analyzed but not used as a direct measure of program quality in our annual program review score card.

Post-NRMP survey. An anonymous survey is sent to all applicants ranked high enough to match who chose another program. The survey asks this group of applicants to respond to questions about overall program quality, program structure, interviews with faculty and residents, stipends and benefits, spouse/significant other opportunities, location, diversity, proximity to family, lifestyle issues, opportunities for off-campus rotations and technology support. The results of this survey are analyzed and included in the annual program review. Although some factors are impossible to change (e.g. location of program), all other issues identified are addressed in an action plan and follow-up report. Results of the survey are compared year-to-year.

### Exit Interviews

The program director conducts exit interviews with each resident or fellow near program completion. The results of these interviews are used to identify opportunities for program improvement. A key metric is the number of residents or fellows who indicate they would choose the program again. These comments are included in narrative form as an addendum to the report card.

### Case Numbers/Experience

The number, type and distribution of cases and procedures recorded by residents and fellows are compared to ABA requirements. We have selected a program goal of exceeding minimum case/procedure experience by at least 20% in every category. If case/procedure experience falls below this standard, an action plan is generated to correct the deficiency, case/procedure numbers are tracked more closely and changes in the resident schedule are made as needed to secure this experience.

### Diversity

The percent of women applicants who apply, are invited to interview, interview, are ranked and match is tracked. These numbers are compared to national averages included in the annual education issue of the Journal of the American Medical Association (JAMA)[7]. Our program goal is to be greater than 80% of the average percent of women enrolled in anesthesiology.

The percent of under-represented minority (URM) applicants who apply, are invited to interview, interview, are ranked and match are tracked. These numbers are compared to national averages included in the annual education issue of JAMA[7]. To assure a meaningful comparison, the definition of URM applicants is the same as that used in JAMA. Our goal is to be greater than 80% of the average percent of under-represented minorities enrolled in anesthesiology.

### Alumni Survey

A survey of alumni who have graduated in the past year is conducted as part of the annual review. The survey asks about program strengths and weaknesses and suggestions for improvement. It specifically asks for subjects/topics about which graduates of the program felt poorly prepared for independent practice. A key metric in this survey asks residents if they would choose the program again and would recommend it to others.

### Program/Rotation/Faculty/Curriculum Evaluation

#### Resident Evaluations

**Figure 1:**
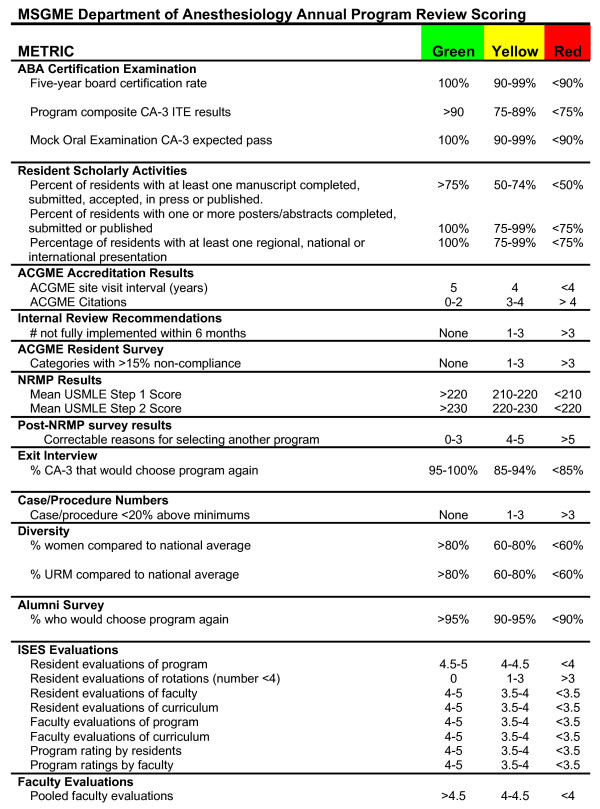
**Color-coded Program Evaluation "Report Card"**.

These key evaluations can be conducted using a variety of instruments. In our program, we use an Integrated Scheduling and Evaluation System (ISES) to collect this information. ISES is a validated, behavioral, electronic evaluation and portfolio tool. Resident/fellow evaluations recorded in ISES are ACGME competency and criterion based, with clear anchors describing each domain of evaluation. ISES evaluations are based on a five-point Likert scale with the capability of reporting mean, median and mode values. Outcomes of resident/fellow evaluations of the program, rotations, faculty and curriculum using ISES are important components of the annual program review. Similarly, ISES is used to obtain faculty evaluations of the program that are required components of the annual program review. Residents and faculty are asked to provide a composite program rating annually to facilitate general assessment of the program that can be compared on a year-to-year basis. Our program established goals for each component of the ISES metrics as detailed in figure 1. Educational initiatives are described in a written action plan if concerns are identified.

#### Faculty Evaluations

Our faculty development process includes review of resident evaluations of faculty that are pooled annually to assure confidentiality and are shared with faculty during a personal annual performance review conducted by the department chair. Collective faculty performance based on ISES evaluations is tracked year to year with a goal of improving on an annual basis. Faculty participation in institutional, regional and national development activities is also monitored and is discussed during the annual performance review of each faculty conducted by the department chair.

Although qualitative and therefore not specifically included in the metrics, written comments are solicited for items in which they may add value. As is true with resident and faculty evaluation, the comments often provide great value in guiding program change and improvement. In our annual review, comments provided by faculty, residents and fellows are listed and reviewed. The comments often stimulate program change/improvement in areas not easily measured numerically.

A composite summary report, including action plans to address areas of concern, is generated in which metrics are compared year-to-year.

As described previously, a "report card" can be used to evaluate the quality of residencies and fellowships that is useful for high-level review[2,4-6]. We describe a "report card" format and assign color coding (red, yellow and green) based on pre-established standards that is used as a major component of the ACGME-required annual program review process. The goals and standards presented apply to the anesthesiology residency at Mayo Clinic Rochester. However, standards for performance can be established in any program based the program's individual characteristics and history in a process in support of continuous improvement.

## Discussion

The annual program review is of value only if action is taken to sustain excellence and/or improve performance. Systematic annual review of anesthesiology residency and fellowship programs requires identification and assessment of broad measures of program quality. We describe an annual program evaluation that meets ACGME requirements, focuses attention on areas for improvement, requires action plans to address these concerns, tracks performance trends year-to-year and guides trainees and faculty in ongoing formative program assessment and program quality improvement. The program "report card" we describe has the advantage of being reproducible and is valuable for tracking performance year-to-year. Color coding provides a high-level assessment of program performance at a glance. There are also several limitations to the annual program review method we describe. The results we report are from only one program and only one specialty. The values assigned to the metrics are arbitrary as they are based on the authors' opinions, historical program controls and the results of using the "report card" methodology in other specialties within Mayo School of Graduate Medical Education. The report card is not comprehensive and other metrics of program quality may be excluded. However, many of the components of our program review could be useful in the evaluation of other anesthesiology programs and they might also be effectively applied to programs in different specialties.

## Conclusions

An annual program review is required to assess all ACGME-accredited residency and fellowship programs to monitor and improve program quality. We describe an annual review process based on metrics that can be used to focus attention on areas for improvement and track program performance year-to-year. A "report card" format is described as a high-level reproducible tool to track educational outcomes over time.

## Competing interests

The authors declare that they have no competing interests.

## Authors' contributions

SR and TL contributed equally in the creation of the "report card" tool and preparation of the manuscript. Both authors read and approved the final manuscript.

## Pre-publication history

The pre-publication history for this paper can be accessed here:

http://www.biomedcentral.com/1472-6920/10/13/prepub
